# Navigating Antimicrobial Resistance Insights: An In-Depth Analysis of Healthcare Providers’ Knowledge, Attitudes, and Practices, with an Emphasis on Precision Medicine in Pakistan

**DOI:** 10.3390/antibiotics14121281

**Published:** 2025-12-18

**Authors:** Sidra Shahid, Aiman Athar, Shahzeen Farooq, Madena Yahya, Muhammad Saad Ashraf, Shafaq Mahmood, Abdul Momin Rizwan Ahmad

**Affiliations:** 1 Department of Humanities and Social Sciences, Pubic Health Cluster, Bahria University, Islamabad 44000, Pakistan; 2 BMY Health, Lahore 54000, Pakistan; aiman.athar945@gmail.com (A.A.); shahzeen.farooq@ucalgary.ca (S.F.); saadashraf292@gmail.com (M.S.A.); shafaq.mahmood@adelaide.edu.au (S.M.); 3 Department of Human Nutrition and Dietetics, NUST School of Health Sciences, National University of Sciences & Technology (NUST), Sector H-12, Islamabad 44000, Pakistan; 4 Department of Health Sciences, University of York, York YO10 5DD, UK

**Keywords:** knowledge, attitude, practices (KAP), antimicrobial resistance (AMR), precision medicine (PM), healthcare professionals (HCP), thematic analysis, bivariate analysis, low and middle income countries (LMICs)

## Abstract

Antibiotics play a crucial role in the treatment of many complicated problems in clinical medicine, but antimicrobial resistance (AMR) has emerged as a serious concern threatening to undermine its effectiveness. Precision medicine (PM) which tailors treatment to individual and genetic and lifestyle factors, may offer a novel approach to combat AMR. Yet, little is known about how healthcare providers in Pakistan understand and integrate the concept of precision medicine within their knowledge, attitudes, and practices (KAP) towards AMR. This study aims to investigate the knowledge, attitude, and practices of healthcare professionals towards AMR and to explore their perception about precision medicine as a strategy to reduce AMR. A mixed method approach was employed for the study. The knowledge, attitude, and practices (KAP) of healthcare professionals (N = 326) were assessed through a validated questionnaire. SPSS version 26 (Statistical Package for the Social Sciences) was employed for descriptive and bivariate analyses to determine KAP score and its association with demographics. Qualitative data were gathered through a focus group discussion and thematic analysis was performed to explore the perception about PM. Results showed that healthcare professionals demonstrate poor knowledge (55.5%), relatively positive attitudes (54.6%), and that nearly half had unfavorable practices (48.3%). Significant associations (*p* < 0.05) were found between KAP scores and factors such as location, healthcare setting, educational level, professional designation, and prior infectious disease training. During focus group discussion (FGD), HCPs emphasized the cautious use of PM, particularly in the management of resistant infections. However, limitations in resources, poor governance, poverty, and access to data and testing facilities were highlighted as barriers in the implementation of PM into practice. This study highlights critical gaps in knowledge and practices towards AMR among HCPs. While PM is viewed as a potential tool against AMR, systemic support, resource allocation, and targeted awareness programs are essential to integrate PM into clinical practice.

## 1. Introduction

Antibiotics provide a paradigm shift in medicine to treat many complicated problems in clinical medicine, but antimicrobial resistance (AMR) has not only hampered the process but has also reversed some progress [1]. According to WHO, antimicrobial resistance (AMR) can result in higher mortality, greater morbidity, and longer hospital stays [2]. It has affected every area of health and is impacting all of society. Moreover, the current rise in the number of AMR cases has posed serious challenges to our ability to treat infections [3].

AMR has been declared a silent pandemic, especially in developing countries, and healthcare professionals (HCPs) have an important role to play. One of the five major objectives of the global action plan on AMR [4] is ‘to improve awareness and understanding of AMR through effective communication, education and training’. It is also evident that the knowledge, attitudes, and perceptions (KAP) towards AMR of all the stakeholders, including healthcare professionals, act as an indicator that helps us to determine the willingness to counter AMR [5]. A recent study in Pakistan showed that the knowledge of clinicians is relatively poor regarding AMR [6]. Another study carried out among HCPs in Zambia showed that a high AMR knowledge score among HCPs was positively correlated with a positive attitude towards combating AMR and thus a lower incidence of AMR [7]. Such studies help uncover the gaps and guide the development of targeted interventions. Non-compliance with prescribed medication has been identified as a significant factor in the development of AMR [8]. According to a study, this issue was further exacerbated during COVID-19 when, due to the vague nature and lack of research on COVID-19, the use of broad-spectrum antibiotics actually led to an increase in AMR, which could have been prevented if the medicine prescribed had been specific to the virus [9]. An emerging application of precision medicine (PM) addresses the globally increasing prevalence of AMR. PM is an approach to treating and preventing diseases that is tailored to the genetic, environmental, and lifestyle factors of each individual patient [10]. It reinforces the idea that more accurate understanding of disease mechanisms is the key to enhancing therapeutic treatment [11].

Recent advancements in genetic and other molecular techniques are enabling clinicians to discover rapid diagnoses and access timely information to guide their prescribing practices [12]. This approach will not only lead to quick treatment but will also assist in the appropriate prescribing of antibiotics, which is one of the major causes of antibiotic resistance [13]. By using molecular profiling techniques, like genetic testing for drug related genes, decisions by healthcare professionals will have well-grounded confirmation to provide appropriate treatment to each patient [14]. In another study, it has been reported that modern and personalized antibiotic therapy based upon a patient-specific microbiome effectively enhanced the effectiveness of the treatment [15]. Numerous studies have shown positive clinical outcomes by using PM, particularly in sepsis [16] and pneumonia.

In Pakistan, some studies have been carried out to evaluate the KAP of healthcare professionals regarding AMR and a few studies have been found on the use of genomics and precision medicine from the perspective of oncology, but no such comprehensive research has been performed so far to study the use of precision medicine in relation to AMR. Before the integration of precision medicine into healthcare practices, it is crucial to conduct an assessment of the current knowledge levels among healthcare providers. Thus, for the implementation of the use of precision medicine in the healthcare settings of Pakistan, it is necessary to equip our HCPs with enough knowledge to practice it.

As this is a novel phenomenon, the present study is specifically designed to test the knowledge, attitude, and practices of healthcare professionals regarding AMR and its interventional strategies. Furthermore, this study also serves as a foundational step to explore the perception of healthcare workers on PM and whether it has the potential to reduce the rise in AMR at the global and national levels.

Addressing AMR requires a multifaceted approach. The One Health concept, integrating human, animal, and environmental health, emphasizes prevention, rational antimicrobial use, and surveillance. Within this framework, assessing healthcare providers’ knowledge, attitudes, and practices is vital for strengthening the human health component in Pakistan.

## 2. Materials and Methods

### 2.1. Operational Definitions

**Knowledge:** The knowledge score was assessed using a weighted marking system, where more difficult questions were assigned higher marks. This approach ensured that respondents who correctly answered more challenging questions received additional points. To categorize the knowledge levels, the mean ± 1 standard deviation (SD) of the total scores was calculated. Respondents scoring above the mean + 1 SD were classified as having “satisfactory” knowledge, while those scoring below the mean − 1 SD were classified as having a “limited” knowledge of antimicrobial use and resistance.

**Attitude:** For attitude-related queries, a Likert scale rating from 1 to 5 was assigned to each response. The total attitude score ranged from 10 to 50, with the mean ± 1 standard deviation (SD) used as the cutoff. Respondents scoring above the mean + 1 SD were classified as having a “favorable” attitude, while those scoring below the mean − 1 SD were classified as having an “unfavorable” attitude towards antibiotic prescription and AMR.

**Practices:** The assessment of practices related to antibiotic prescription was determined by assigning one point for each correctly executed step, resulting in a total score between 10 and 40. The mean ± 1 standard deviation (SD) was used as the cutoff. Respondents with a practice score equal to or exceeding the cutoff value were classified as having “appropriate” practices, while those below this threshold were classified as having “inappropriate” practices.

### 2.2. Study Design and Sampling Techniques

A cross-sectional mixed method design was employed in this study which allowed a thorough assessment of healthcare professionals’ KAP through quantitative surveys, providing numerical insights. Additionally, the qualitative component explored perceptions of precision medicine in combating AMR, capturing nuanced perspectives, experiences, and underlying reasons that may not be fully captured by quantitative measures alone. By combining both approaches, we gained a more holistic understanding of the complex interplay between knowledge, attitudes, practices, and perceptions among healthcare professionals in the context of addressing AMR [17]. It also helped to explore their perspectives towards precision medicine to combat AMR in their practice.

Study population included registered healthcare professionals aged 25–65 years in Pakistan, including general physicians (GPs), interns, postgraduate trainees, consultants, and pharmacists. All registered male and female healthcare professionals in Pakistan (including GPs, interns, postgraduate trainees, consultants, and pharmacists) aged 25–65 years who consented to participate in our study were included. Healthcare professionals who did not meet the inclusion criteria or those who refused to participate were excluded from the survey.

Data were collected over a three-month period, i.e., January 2024 to March 2024, from doctors employed across diverse healthcare settings (both public and private) in Pakistan to enhance the sample’s representativeness, including:

Primary healthcare centers, i.e., Basic Health Units (BHUs) and Rural Health Centers (RHCs).

Secondary healthcare hospitals, i.e., District Headquarter Hospitals (DHQs) and Tehsil Headquarter Hospitals (THQs).

Tertiary care and teaching hospitals (both public and private).

Hospital and community pharmacies.

Family and GP clinics.

For the quantitative survey, the convenience sampling method was used to recruit the participants, whereas purposive sampling was employed to include healthcare professionals for the focused group discussion (FGD). We specifically invited individuals with a minimum of 5 years of experience, ensuring a wealth of expertise. We aimed to include those participants in the FGD who were vocal and actively engaged in discussions. Additionally, our selection criteria prioritized representation from various medical specialties, such as medicine, infectious diseases, gynecology, and a pharmacist. This deliberate approach ensured that the FGD captures insights from experienced and communicative professionals across diverse medical domains, enriching the depth and breadth of our study. This deliberate composition enabled the FGD to capture diverse clinical perspectives and practical insights relevant to AMR management within Pakistan’s healthcare context.

### 2.3. Sample Size

The sample size was calculated using the OpenEpi software (Version 3.01; Open Source Epidemiologic Statistics for Public Health, Emory University, Atlanta, Georgia, USA)sample size calculator [18], utilizing data from a reference study assessing knowledge of AMR among healthcare professionals in West Bengal, India [19]. Assuming a 95% confidence level (Z = 1.96), an anticipated population proportion of 77.3% (*p* = 0.773), and an absolute precision of 0.05, the calculated sample size was approximately 270. This reference is pertinent because it addresses comparable population characteristics, study objectives, and outcome measures related to antibiotic usage and AMR awareness.

Moreover, the number of factors anticipated to influence the study outcomes—including demographic variables (age, education level), behavioral aspects (antibiotic usage patterns), and knowledge levels—were carefully considered. It further guided the identification of these factors, ensuring the sample size was sufficiently powered to detect statistically significant differences and associations.

### 2.4. Data Collection Procedures

The data collection procedure encompassed both quantitative and qualitative components. Prior to the main survey, a pilot survey was conducted with 15–20 participants to refine the tools used for assessing knowledge, attitudes, and practices (KAP) on AMR. Content validity was established through expert review, and internal consistency was confirmed after pilot study (Cronbach’s alpha > 0.7). Informed written consent was obtained from participants, ensuring their understanding of the voluntary nature of participation and the strict confidentiality of their responses.

For the quantitative component, a self-administered data collection technique was employed to gather information from the HCPs. An estimated 15–20 min was required for completion of the questionnaire. Special attention was given to reach healthcare professionals in hard-to-access areas, such as BHUs and RHCs. Thus, the questionnaire was also disseminated through email and other online tools to enhance the survey’s representativeness.

In the qualitative component, an FGD was conducted with five participants, lasting approximately 45 to 60 min using a semi-structured interview guide. The discussion was recorded and transcribed into a digital format. It addressed various aspects related to precision medicine and its role in minimizing AMR, including understanding precision medicine, current perceptions of AMR, and the integration of precision medicine in practice. Thematic analysis was employed to identify sub themes and capture the diverse perspectives of healthcare professionals with extensive experience.

### 2.5. Data Collection Tool

The KAP questionnaire has been adapted from various sources in the literature and tailored to the local context [6,20,21,22,23].

It consists of three sections, each addressing knowledge, attitudes, and practices, alongside socio-demographic information. The questionnaire employed both closed-ended questions, utilizing a 5-point Likert scale (ranging from “strongly agree” to “strongly disagree”), and 3-point Likert scale questions (“Yes”, “No”, “Not sure”). The first section gathered information on socio-demographics and professional and academic background.

The second section comprised 10 questions assessing participants’ knowledge regarding AMR. The third section focused on participants’ attitudes, presenting 10 questions related to antimicrobial use and resistance. The final section examined practices with 10 questions concerning antibiotic prescription habits.

A qualitative questionnaire, adapted from a prior study [24], supplements the quantitative approach (Supplementary Materials). This qualitative section probed into participants’ knowledge, accessibility, and perceived benefits of precision medicine.

### 2.6. Statistical Methods

Data were analyzed using descriptive statistics (frequencies, percentages, means, and medians, as appropriate) to summarize participants’ knowledge, attitudes, and practices (KAP). Associations between demographic characteristics and KAP scores were assessed using the chi-square test. A *p*-value < 0.05 was considered statistically significant. The overall response rate was 89%. Item non-response was minimal (<5%); therefore, cases with missing data were excluded from the analysis (complete-case analysis), and no imputation was performed.

### 2.7. Ethical Consideration

Data were collected after obtaining informed written consent, signed by the participant. Ethical approval was obtained from the Ethical Review Committee (ERC) of BMY Health (Protocol number BMY-ERC2-2024) prior to data collection.

## 3. Results

### 3.1. Descriptive Findings

The current study included 326 healthcare professionals with a higher percentage of female participants (58.5%) as compared to the male participants (41.2%). Table 1 summarizes the socio-demographic characteristics of the study participants. The majority of our study sample was aged between 24 and 35 years (31.4%) and 36 and 45 years (29.3%). Participants were mainly from major cities including Karachi (35.7%), Islamabad/Rawalpindi (34.5%), and Lahore (23.2%), with a small percentage from other cities (6.4%) (Table 1).

A significant proportion of participants had a bachelor’s degree (49.1%), with many also holding higher specializations, such as clinical fellowships (FCPS) (26.5%), master’s degrees (10.1%), and doctorates (15%). Notably, a large proportion had 1–5 years of work experience (47.3%). The majority were medical officers (27.1%), followed by consultants (25%) and pharmacists (14%). Most participants were not specialists (68.1%), and a significant majority lacked training in microbiology (90.2%). Only 21.7% of participants were involved in antimicrobial stewardship programs at their healthcare settings (Table 1).

Table 2 shows that participants had the highest mean score in attitude, followed by knowledge and practice, indicating positive attitudes but limited knowledge and suboptimal practices.

**Figure 1:**
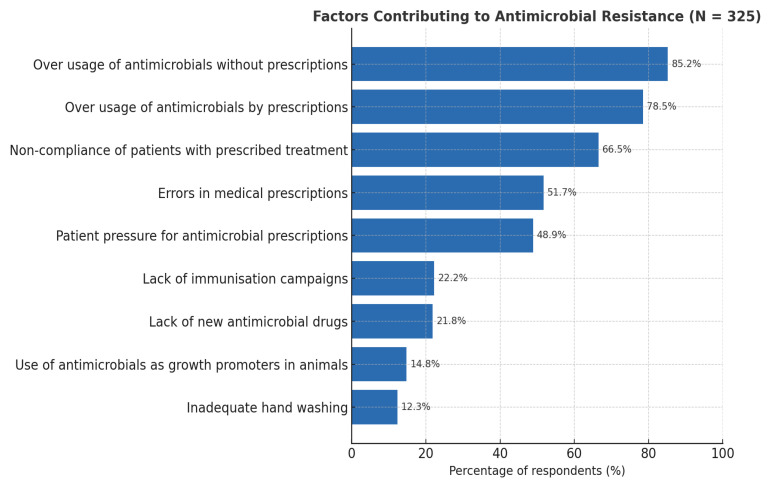
Factors selected by the healthcare professionals to be the most contributory towards antimicrobial resistance (N = 325).

Figure 1 highlights the factors most identified by healthcare professionals as contributing to antimicrobial resistance. The leading contributors include overuse of antimicrobials without prescriptions and overuse by prescription, with 85.2% and 75.8%, respectively.

Percentages for contributory factors were calculated as the number of respondents selecting each option divided by the total sample size (N = 325), multiplied by 100.

**Figure 2:**
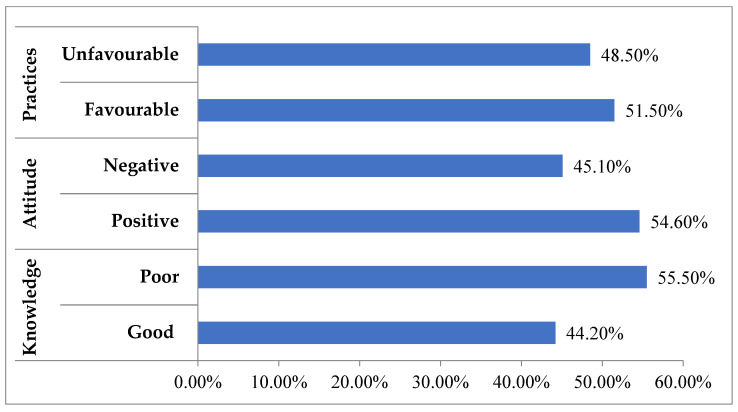
Distribution of healthcare professionals’ knowledge, attitudes, and practices towards antimicrobial resistance (N = 325). Classification into categories was based on scoring criteria defined in the Methods section (Operational Definitions).

Our study indicates that 55.50% of HCPs reported having poor knowledge, while a significant proportion (54.60%) demonstrated a positive attitude. In terms of practices, 59.9% displayed favorable behaviors, although 48.5% still exhibited unfavorable practices (Figure 2).

### 3.2. Bivariate Analysis

Table 3 and Table 4 summarize the findings of chi-square analysis to assess the relationship between socio-demographic variables and the knowledge, attitudes, and practices of healthcare professionals in Pakistan regarding antimicrobial use and resistance.

The analysis identified significant associations between knowledge levels and specific socio-demographic factors, including location, healthcare setting, and professional designation (*p* < 0.05). Similarly, attitudes were also found to be significantly associated with location, work setting, educational level, and years of practice (*p* < 0.05). Practices were also significantly influenced by location, healthcare setting, educational level, designation, and prior training in infectious diseases (*p* < 0.05).

### 3.3. Focus Group Discussion

The focus group discussion (FGD) included five participants with diverse medical roles. Among the participants, three were female, comprising a medical officer, a gynecologist, and a surgeon. the remaining two participants were male, including a pharmacist and a medicine specialist. This varied group provided a range of perspectives from different areas of the medical field. These respondents were coded as R1, R2–R5, respectively. During thematic analysis, seven themes and 16 sub themes were identified. The findings of the qualitative survey are summarized in Table 5.

According to the respondents, the most significant factors contributing to AMR were found to be cultural and religious practice, self-medication, overuse and misuse of antibiotics, and hygiene practices; however, these views reflect self-reported opinions and were not supported by species-specific or antibiotic-level data in this study. Many healthcare professionals recognized precision medicine as a promising solution to AMR, although governance and poverty hinder the adoption of innovative approaches. They emphasized the need for more research on genetics and mass testing for the general population, as well as providing training for healthcare providers to incorporate precision medicine into their practice to combat AMR. A consultant particularly emphasized the role of precision medicine in treating resistant cases.

## 4. Discussion

This study highlights significant gaps in the KAP of healthcare professionals regarding AMR. While 54.6% of participants exhibited a positive attitude towards combating AMR, more than half (55.5%) displayed poor knowledge and nearly half (48.5%) engaged in unfavorable practices. These findings underscore the need for targeted educational interventions to improve knowledge and practices, despite the generally positive attitudes.

The socio-demographic characteristics of the study participants showed a wide range of diversity in the healthcare providers who participated in this study. Understanding this is vital for interpreting the study’s findings and for designing targeted interventions in healthcare to help combat antimicrobial resistance. The significant representation of younger participants with 31.4% belonging to the age group 24–35 represents an increasing trend towards a more dynamic and adaptable workforce, where new policies and strategies can be implemented. A total of 49.4% said that they do not know about antimicrobial stewardship programs (AMS) which is concerning as AMS programs are essential to combat AMR and ensure rational use of antibiotics in healthcare settings. One reason could be a lack of finances and leadership in hospitals to initiate the program. Pakistan’s national action plan (2017–2022) has recognized antimicrobial stewardship as a priority; however, its implementation is a challenge and still remains inconsistent across different healthcare settings [25]. It also requires a team leader with enough resources provided by hospital administration for implementation [26].

On the other hand, there seems to be a large discrepancy between the effectiveness of international policies and local policies in the prescription of antibiotics, with 32.9% of participants strongly agreeing that international policies take precedence. This finding suggests that there may be a tendency among healthcare professionals to prioritize global or international guidelines over local or national ones, despite the fact that national guidelines are more directly applicable to their daily practice. A study in Zimbabwe [26] reported that national guidelines were the main source for guiding prescribing in routine practice (93%). It further emphasizes the need for a balance between international and national guidelines. While international policies provide a useful overarching framework, it is crucial that national guidelines be developed and strengthened.

This study reported that 55.50% of HCPs presented to have poor knowledge about AMR. This is in consistency with some of the previous studies of Pakistan [27] and slightly in contrast with global studies [28,29,30]. It suggests that there is a difference in healthcare infrastructure, access to continuous medical education, and resource allocation between countries. Moreover, limited formal training on antimicrobial resistance and stewardship, lack of continuous professional development opportunities, and insufficient integration of AMR-related content into medical and allied health curricula in Pakistan are also key factors leading to poor knowledge among healthcare professionals [31]. However, this score reflects a potential gap in knowledge that can be improved with the implementation of interventions, stewardship initiatives, or educational campaigns aimed specifically at reducing those gaps.

Overall, the respondents demonstrated a positive attitude, as shown by the mean and median scores below. A study in Nigeria showed a similar result, with 79% of HCPs recorded as having a positive attitude towards proper antibiotic prescription, while the results also showed consistency with a local study conducted in the KP province which reported good attitudes of HCPs towards antibiotics and its use [32,33]. It showed that attitudes mostly remained positive but limited translation into practice was reported, which could be due to the gap between intention and execution. While HCPs may recognize the importance of rational antibiotic use and express a positive attitude, their ability to apply this in practice could be hindered by limited knowledge or lack of resources. Inadequate access to continuous medical education or insufficient training in microbiology may leave them without the necessary skills to implement antimicrobial stewardship effectively.

Figure 1 highlights the factors identified by healthcare professionals as contributing to antimicrobial resistance. Evidence indicates that behavior change interventions, including education, training, guideline enforcement, audit and feedback, and restricted prescription, have been shown to reduce antimicrobial use and improve guideline adherence in multiple settings, particularly in LMICs [34].

Additionally, constraints, such as the availability of diagnostic tools, limited institutional support, or overwhelming patient loads, may further restrict their ability to practice what they know is best. Such a disconnect between attitude and practice underscores the need for not just awareness rising but also capacity building interventions that equip HCPs with the tools and resources that they need to act on their positive attitude. Participants exhibited slightly low levels of compliance to practices (59.60%) which is also consistent with the local studies [32], while in contrast in global studies reported in Ethiopia and Egypt HCPs showed good practices [35,36]. However, the scores in the present study reflect a potential lack of consistency in compliance which can be improved or optimized with the recognition of barriers, like strict compliance to AMS in hospitals and better infrastructure and resources.

Though a good attitude was found in our study, poor knowledge and practices pose concerns to the increasing risk of antimicrobial resistance in the country. Low- and middle-income countries not only lack in the implementation of antimicrobial stewardship programs in their hospitals, but they also lack in sufficient data [35]. Thus, there is a dire need to implement a uniform policy to cater to this serious risk at a national level.

Years of practice and level of education were also found to have significant associations with attitude and practices. Some previous studies [28,37] also reported an association of level of education and practices of healthcare professionals. One reason could be that less experienced HCPs may exhibit more positive attitudes towards AMR due to recent training or enthusiasm, but their practices might not yet reflect this. Conversely, those with more experience may have had more opportunities to integrate their knowledge into effective practices over time. Our findings highlight gaps in healthcare providers’ KAP towards AMR, reinforcing the need for a One Health approach that integrates prevention, prudent use of antimicrobials, and surveillance. Strengthening the human health pillar is therefore essential for operationalizing this framework in Pakistan.

Focus group discussions (FGDs) not only gave deepened points of view but also helped to obtain insights from professionals working in the field. FGDs are considered as a vital source for collecting qualitative data [38]. Different perspectives from various domains enriched the discussion and contributed to the existing literature. Cultural and religious practices, self-medication, and overuse and misuse of antibiotics were identified as major challenges of AMR, which is also evident from a previous study [39]. Another study highlighted that sociocultural determinants, such as patient demand, self-medication, and easy access to non-prescription antibiotics, significantly drive misuse and overuse. Although refusal of medications due to religious beliefs is recognized in some communities, no published data currently quantify its prevalence in Pakistan [40]. Religious and cultural beliefs have been shown to influence hygiene and infection-control behaviors (for example, hand hygiene and avoidance of physical greetings) in healthcare and community settings [41,42].

Community engagement, prevention at large, and precision medicine were identified as imminent solutions to the growing challenge of antimicrobial resistance. Creating awareness among common people and bringing physicians, veterinarians, and environmentalists on one platform to solve this problem has proven to be very effective. Healthcare professionals emphasized that precision medicine powered by AI can prove to be a revolutionary approach to cater to AMR, especially resistant cases, in healthcare settings. A study pointed out that genomic data in a knowledge-generating healthcare system infrastructure provide excellent opportunities to utilize the full potential of that information to optimize patient care [43]. Precision medicine has huge potential to perform in the world of technology and artificial intelligence. According to WHO’s report on artificial intelligence in health in 2021, AI can support precision medicine by enabling tailored treatment regimens through the analysis of genetic and clinical data [44].

Some of the barriers identified in implementing PM include weak governance and widespread poverty. As Pakistan is a low- and middle-income country (LMIC), these challenges raise significant concerns for the effective adoption of PM. The budget is limited for healthcare and access to data and testing facilities is also inadequate. Thus, a robust healthcare system and timely interventions are essential to address the challenges of AMR and to effectively leverage PM in combating it. Clinical microbiology laboratories in major hospitals generally provide pathogen identification and antimicrobial susceptibility testing, but the integration of patient genetic data and pharmacogenomics into treatment decisions is rare and largely research-oriented rather than standard clinical practice. With digital health initiatives growing in Pakistan, the integration of PM is increasingly feasible. It can bridge the diagnostic gap and support task shifting, particularly in low resource settings.

Some policies are documented by DRAP on AMR, like “Guidelines on responsible use of antimicrobials in human health” [45], but their implementation is still a challenge. Some of the highlighted points in these guidelines include antibiotics stewardship guidelines and the regulation of antibiotics sale. In addition to that, DRAP is also involved in surveillance and supports the national action plan on AMR. However, precision antimicrobial approaches are not yet a part of national implementation and the discussion in this study therefore presents personalized medicine as a conceptual future direction, aligned with Pakistan’s existing AMR priorities.

### Recommendations

Healthcare professionals are at the forefront for managing antibiotics; thus, it is essential for them to upgrade their knowledge related to antimicrobials, AMR, and antimicrobial stewardship (AMS) [20]. To enhance the use of precision medicine and raise awareness among healthcare professionals (HCPs), it is essential to address the challenges they face. This can be achieved by offering ongoing education, integration of AMR and precision medicine in national clinical protocols, and comprehensive training to overcome obstacles, such as improving governance and strengthening policy implementation. As this study was conducted in urban cities of Pakistan, more studies in rural areas can provide better insights where resources and infrastructure are serious issues.

This study has several limitations. Firstly, it was conducted only in selected cities of Pakistan, which may limit the generalizability of the findings to doctors practicing in rural or underserved areas. Additionally, the voluntary nature of participation might have introduced selection bias, as doctors more interested or knowledgeable about antibiotic stewardship and AMR could have been more likely to respond. Since the data are self-reported, social desirability bias may have influenced responses, potentially leading to an overestimation of appropriate knowledge, attitudes, and practices. The cross-sectional design captures information at a single point in time, restricting the ability to assess changes in prescribing behavior or knowledge over time. Moreover, the study did not include objective measures, such as prescription audits, to validate reported practices. Finally, external factors influencing antibiotic prescribing, such as institutional policies or patient demand, were not explored, which may affect the comprehensiveness of the findings. Although a few questions on the choice of antibiotics for certain diseases were included, the study did not comprehensively address pathogen–antibiotic associations or the full range of antibiotic families prescribed.

Our questionnaire did not include items assessing knowledge of genetic or molecular mechanisms (e.g., plasmids or resistance genes, such as *blaNDM* or *blaCTX-M*); thus, this aspect was not evaluated and should be considered in future studies and training initiatives. The study did not assess knowledge or attitudes regarding clinical microbiology laboratory practices, which could be explored in future research. Moreover, this study attempted to control for major confounding factors, some potential confounders could not be addressed due to limited time, resources, and data availability. Future studies with larger samples and more comprehensive data collection are recommended to incorporate these variables and better account for residual confounding.

## 5. Conclusions

This study revealed that HCPs working in different cities of Pakistan exhibited a good attitude but poor knowledge and unfavorable practices while using antibiotics. AMR poses a significant global threat and needs a multidisciplinary approach to ensure the rational use of antibiotics. The findings of the study emphasized the urgent need to enhance knowledge, attitude, and practices, particularly in LMICs where AMR continues to rise. To address these gaps, effective and robust continued education and training are required to improve the knowledge, attitude, and practices of HCPs. Though Pakistan has a national action plan on AMR that has an important clause to create awareness and change the behavior, its strict implementation is needed at primary, secondary, and tertiary levels.

During the focus group discussion (FGD), precision medicine emerged as a promising strategy in addressing antimicrobial resistance (AMR). Participants highlighted the importance of raising awareness among healthcare professionals and the public about the potential of precision medicine in combating AMR. Moreover, it also emphasized genetic research and expanding mass testing. Effective governance at both the national and provincial levels is crucial for creating a cohesive strategy, ensuring that precision medicine supported by artificial intelligence is integrated into healthcare systems, and addressing challenges, such as access to advanced diagnostics and treatments.

## Figures and Tables

**Table 1:** Socio-demographic characteristics of the study participants (N = 326).

Gender	Male	191	58.5
	Female	135	41.3
Age (years)	24–35	103	31.4
	36–45	96	29.3
	46–55	86	26.2
	56–65	41	12.5
Location	Karachi	116	35.7
	Islamabad/Rawalpindi	113	34.5
	Lahore	76	23.2
	Others	21	6.4
Work setting	Public sector	176	54.3
	Private sector	150	45.7
Healthcare setting	Primary healthcare (BHU, RHC)	20	6.1
	Secondary healthcare (DHQ, THQ)	52	16.2
	Tertiary healthcare	191	58.2
	Family/GP clinic	25	7.6
	Pharmacy	38	11.6
Educational level	Bachelor’s degree	161	49.1
	Master’s degree	33	10.1
	FCPS	86	26.5
	Doctorate	46	14.1
Years of practice	<1 year	109	33.4
	1–5 years	154	47.5
	6–10 years	31	9.5
	>10 years	32	9.8
Designation	Medical officer	89	27.3
	Postgraduate trainee	74	22.7
	Consultant	82	25.2
	Pharmacist	45	14.1
	Assistant/Associate professor	36	11.0
Specialist	Yes	141	68.1
	No	66	31.9
Antimicrobial stewardship program	Yes	74	21.7
	No	156	45.9
	Do not know	96	29.4
Training in microbiology/infectious diseases	Yes	31	9.5
	No	295	90.5

**Table 2:** Mean scores for knowledge, attitude, and practices among the study participants.

Domain	Mean Score ± SD
Knowledge	16.04 ± 4.004
Attitude	24.99 ± 3.900
Practice	14.64 ± 2.988

**Table 3:** Relation between socio-demographic characteristics and level of knowledge among healthcare professionals of Pakistan regarding antimicrobial use and resistance. N = 326.

Demographic Characteristics	Poor Knowledge n (%)	Good Knowledge n (%)	*p*-Value
Gender			0.876
Male (n = 134)	53 (39.3)	81 (60.7)	
Female (n = 192)	73 (38.2)	119 (61.7)	
Age (years)			0.060
25–35	52 (50.5)	51 (49.5)	
36–45	48 (50.5)	47 (49.5)	
46–55	50 (58.1)	36 (41.9)	
56–65	30 (73.2)	12 (26.8)	
Location			0.004 *
Karachi	53 (45.3)	64 (54.7)	
Islamabad/Rawalpindi	62 (54.9)	51 (45.1)	
Lahore	54 (72.0)	21 (28.0)	
Others	12 (57.1)	9 (42.9)	
Work setting			0.846
Private sector	83 (55.7)	66 (44.2)	
Public sector	98 (55.36)	79 (44.63)	
Healthcare setting			0.001 *
Primary (BHU, RHC)	11 (55.0)	9 (45.0)	
Secondary (DHQ, THQ)	33 (62.3)	20 (37.7)	
Tertiary	107 (56)	84 (44)	
Family/GP clinic	13 (54.2)	11 (45.8)	
Pharmacy	17 (44.7)	21 (55.3)	
Educational level			0.357
Bachelor’s degree	90 (55.9)	71 (44.1)	
Master’s degree	15 (45.5)	18 (54.5)	
FCPS	53 (61.6)	33 (38.4)	
Doctorate	23 (50.0)	23 (50.0)	
Years of practice			0.545
<1 year	60 (55.0)	49 (45.0)	
1–5 years	83 (53.5)	72 (46.5)	
6–10 years	21 (67.7)	10 (32.3)	
>10 years	17 (54.8)	14 (45.2)	
Designation			0.004 *
Medical officer	36 (40.4)	53 (59.6)	
Pharmacist	31 (67.4)	15 (32.6)	
Postgraduate trainee	40 (52.6)	34 (47.4)	
Assistant/Associate professor	26 (72.2)	10 (27.8)	
Consultant/Registrar	48 (59.3)	33 (40.7)	
Specialist			0.731
Yes	67 (56.8)	51 (43.2)	
No	114 (54.8)	94 (45.2)	
Average patients/day			0.451
≤25	44 (49.0)	55 (51.0)	
26–50	66 (51.0)	61 (49.0)	
>50	60 (51.0)	40(52.6)	
AMS program			0.370
No	82(52.56)	74(47.43)	
Yes	40 (54.1)	34 (45.9)	
Do not know	59 (61.5)	37 (38.5)	
Training (microbiology/ID)			0.382
No	161 (54.6)	134 (45.4)	
Yes	20 (64.5)	11 (35.5)	

Note: * *p* < 0.05 indicates statistically significant difference.

**Table 4:** Relation between socio-demographic characteristics, level of attitude, and practices among healthcare professionals of Pakistan towards antimicrobial use and resistance. (N = 326).

Socio-Demographic Characteristics	Positive Attitude n (%)	*p*-Value	Favorable Practice n (%)	*p*-Value
Gender				
Male	109 (51.9)	0.430	93 (49.3)	0.911
Female	70 (56.8)		66 (48.4)	
Age (years)				
25–35	51 (49.5)	0.546	41 (39.8)	0.400
36–45	55 (57.3)		44 (46.3)	
46–55	51 (59.3)		49 (57.0)	
56–65	22 (53.7)		25 (61.0)	
Location				
Karachi	87 (74.4)	0.000 *	76 (65.0)	0.000 *
Islamabad/Rawalpindi	46 (40.7)		45 (39.8)	
Lahore	34 (44.7)		32 (42.1)	
Others	12 (57.1)		6 (30.0)	
Work setting				
Public sector	71 (47.3)	0.014 *	88 (49.7)	0.739
Private sector	108 (61.0)		71 (47.7)	
Healthcare setting				
Primary (BHU, RHC)	13 (65.0)	0.477	10 (50.0)	0.009 *
Secondary (DHQ, THQ)	25 (47.2)		16 (30.2)	
Tertiary	110 (57.6)		98 (51.6)	
Family/GP clinic	12 (48.0)		18 (44.7)	
Pharmacy	19 (57.6)		17 (51.6)	
Educational level				
Bachelor’s degree	80 (49.7)	0.040 *	76 (47.2)	0.056
Master’s degree	57 (65.5)		16 (34.8)	
FCPS	21 (45.7)		16 (48.5)	
Doctorate	21 (63.6)		51 (59.3)	
Years of practice				
<1 year	70 (64.2)	0.034 *	65 (59.6)	0.002 *
1–5 years	72 (46.5)		59 (38.1)	
6–10 years	18 (58.1)		15 (48.4)	
>10 years	19 (59.4)		20 (64.5)	
Designation				
Medical officer	42 (47.2)	0.100	32 (36.0)	0.020 *
Postgraduate trainee	50 (67.6)		27 (58.7)	
Consultant/Registrar	45 (54.9)		43 (58.1)	
Pharmacist	25 (54.3)		20 (57.1)	
Assistant/Associate professor	17 (47.2)		37 (45.1)	
Specialist				
Yes	111 (53.1)	0.488	103 (47.9)	0.818
No	68 (61.3)		56 (49.3)	
Patients seen/day				
≤25	78 (56.9)	0.758	66 (48.5)	0.979
26–50	59 (50.0)		54 (49.5)	
>50	42 (51.9)		39 (52.6)	
AMS program				
Yes	87 (70.7)	0.794	85 (54.1)	0.100
No	38 (35.2)		29 (39.2)	
Do not know	54 (56.3)		45 (47.4)	
Training (microbiology/ID)				
Yes	12 (38.7)	0.087	9 (29.0)	0.021 *
No	167 (56.4)		150 (50.8)	

Note: * *p* < 0.05 indicates statistically significant difference.

**Table 5:** Key identified themes.

Themes	Sub Themes	Quotations
1. AMR challenges	Cultural and religious practices Self-medication, overuse and misuse of antibiotics Hygiene practices	a lot of people have religious beliefs that prevents them from adopting behaviors that might curb infections like we will not stop shaking hands, we will not stop hugging people, because this goes against our cultural and religious norms. It’s a norm in our country, that if you have prescribed an antibiotic to one person, and if another person in that household gets an infection or something, the first person who has had the antibiotic they will kind of self-prescribe. we have to prescribe antibiotic for every infection even post operatively as it is our patients whose hygiene is not good and sometimes equipment used are not sterilized too
2. Solutions to AMR	Prevention at community level Immunization PM—a new approach	Immunization campaigns should run rigorously it would eventually prevent us from prescribing all these antibiotics and in longer run prevent antimicrobial resistance precision medicine keeps a very holistic approach for AMR, as I said to a person, their behavioral health, their exposures, their genetics, and their character
3. Precision medicine	Perception about precision medicine	what’s needed should only be prescribed tailoring an individual’s medication requirement to everything healthy, taking a more holistic approach the right medicine for the right indication
4. Integration of PM into practice	Genetic research and mass testing Training of healthcare providers	first of all, you need to conduct research on finding out what those genetic needs are and what those genetic markers are and then developing something that is tailored to those particular markers We have no culture of involving the infectious disease or microbiologist, much can be achieved through continuous training in microbiology and prescribing according to the needs of patients.
5. AMR Challenges and Precision Medicine Solutions	Standardized practice in both government and private sector hospitals Awareness among masses Use in resistant cases	very good example is a hospital setups especially those which are having accreditation with the international regulatory authorities or organizations like JCI where they have guidelines and strict rules that there are certain drugs which can’t be prescribed by everyone we can tackle this issue by educating ourselves and our physicians and public as well If medicines are misused, specifically antibiotics then this could be very alarming if we can take examples there are lots of medicine which have become resistant So, in coming time, it’s really important to understand the concept of precision medicine.
6. Barriers to PM in practice	Governance/poverty Access to data and testing facilities Implementation	The vast majority of our population falls below poverty line. So how is all of this going to work? very tiny amount of our GDP is spent on health care here comes ethical or equity key questions, which needs to be answered If you have access to particular healthcare services, you can gain those benefits that come with precision medicine, but because you are disadvantaged or underprivileged, you cannot access the same level of health care”
7. Facilitators	Educating patients as well as HCPs Policy and legislation	Most of the patients do not adhere to the prescription which needs monitoring and follow ups. Prevention obviously is the answer to everything. We need to make policies for antibiotic prescribing and implement them strictly. Certain rules and regulation in practice and definitely we will move toward precision medicine.

## Data Availability

Complete data are available upon request to the authors.

## Supplementary Materials

The following supporting information can be downloaded at: https://www.mdpi.com/article/10.3390/antibiotics14121281/s1.
