# Supplementary material for: Navigating Antimicrobial Resistance Insights: An In-Depth Analysis of Healthcare Providers’ Knowledge, Attitudes, and Practices, with an Emphasis on Precision Medicine in Pakistan
Source: Antibiotics (Basel). 2025 Dec 18;14(12):1281. doi: 10.3390/antibiotics14121281 (PMC12729868; doi:10.3390/antibiotics14121281)
Supplement: Supplementary file 1 [file antibiotics-14-01281-s001.zip › antibiotics-3883016-supplementary.pdf]

Navigating Antimicrobial Resistance Insights: An In-Depth Analysis of Healthcare Providers' Knowledge, Attitudes, and Practices, with an Emphasis on Precision Medicine in Pakistan

QUESTIONNAIRE

Name (optional):____________________________

Gender:___________________________________

Age:_____________________________________

Location (city/town):________________________

SECTION 1: SOCIO-DEMOGRAPHICS:

1. What is your highest qualification

★ Diploma

★ Bachelor's degree

★ Master's Degree

★ FCPS

★ Doctorate

★ Supra specialist

★ Others (Please specify_____________________)

2. Are you a specialists in any medical field (Holding a postgraduate qualification in the area)

★ YES Please specify_____________________

★ NO

3. How many years of practice do you have

★ Less than one year

★ 1-5 years

★ 6 - 10 years

★ > 10years

4. Have you received extra/further training in microbiology/infectious diseases?

★ YES

★ NO

5. If YES, was it:

★ 1 year ago

★ 1-3 years. ago,

★ more than three years ago

6. Which department do you work in?

Please specify__________________

What kind of work setting you practice in? (please select)

Public Sector Private Sector

7. In which healthcare setting do you practice?

★ Primary healthcare

★ Secondary healthcare

★ Tertiary healthcare

★ Community Pharmacy

8. What is the usual work load at your health facility?

★ Low

★ Medium

★ High

9. Has your health facility implemented an antibiotic stewardship program in your setup?

- Yes
- No
- Don’t know

SECTION 2: KNOWLEDGE ABOUT ANTIMICROBIAL MEDICINE AND ANTIMICROBIAL RESISTANCE

1. Which of the following statements about antibiotics is accurate?

- ★ ☐ Antibiotics are effective against both bacteria and viruses.
- ★ ☐ Antibiotics are only effective against bacterial infections.
- ★ ☐ Antibiotics are primarily used to treat fungal infections.
- ★ ☐ Antibiotics have no impact on infections.

2. Can antibiotics be effective against all types of bacteria?

- ★ ☐ Yes, antibiotics work against all bacteria.
- ★ ☐ No, antibiotics are selective and may not work against certain bacteria.
- ★ ☐ I'm not sure.

3. What are the potential consequences of over-prescribing antibiotics? (Select all that apply)

- ★ Antibiotic resistance
- ★ Increased risk of side effects
- ★ Disruption of the normal microbiota
- ★ Prolonged recovery time for patients
- ★ Other (please specify)

4 . Which of the following conditions do you think can be treated with antimicrobials? (Multiple choices)

- ★ HIV/AIDS

★ Gonorrhoea

★ Bladder infection or urinary tract infection (UTI)

★ Diarrhoea

★ Cold and flu

★ Fever

★

★

Malaria

Measles

★ Skin or wound infection (3 points)

★ Sore throat

★ Body aches

★ Headaches

5. Antibiotic resistance occurs when your body becomes resistant to antimicrobials and they no longer work as well.

★ Yes

★ No

★ Don't know

6. The efficacy of antibiotic is higher if it is newer or of higher price

★ Yes

★ No (correct answer)

★ Don't know

★

★

7. Please encircle the factors you believe to be the most contributory towards antimicrobial resistance: (you may select multiple) ★ Over usage of antimicrobials by prescriptions

★ Over usage of antimicrobials without prescriptions

Errors in medical prescriptions (dose, duration of use, and choice)

Non-compliance of patients with prescribed treatment

★ Inadequate hand washing

★ Lack of immunization campaigns

★ Lack of new antimicrobial drugs

★ Use of antimicrobials as growth promoters in animals

★ Patient pressure for antimicrobial prescriptions

8. All cases with coughs, colds and sore throats get improved with the use of antibiotics

★ Yes

★ No (correct)

★ Don't know

9. Which one of the following antibiotics has the best activity against anaerobes?

★ Ciprofoxacin

★ Cotrimoxazole

★

★

★ Metronidazole

★ I don’t know

10. Antibiotics resistant bacteria spread easily from person to person:

★ Yes (correct answer)

No

Don’t know

★

★

SECTION 3: ATTITUDE TOWARDS USE OF ANTIMICROBIALS AND AMR

1. Antimicrobial resistance is a serious public health issue in Pakistan?	1. Strongly agree 2. Agree 3. Not Sure 4. Disagree 5. Strongly disagree
2 .By limiting the use of antibiotics, good patient care would be impaired?	1. Strongly agree 2. Agree 3. Not Sure 4. Disagree 5. Strongly disagree

3. I believe that prescribing antimicrobial does not cause any damage when patients don't need them.	1. Strongly agree 2. Agree 3. Not Sure 4. Disagree 5. Strongly disagree
4. We need to establish courses on rational use of antimicrobials?	1. Strongly agree 2. Agree 3. Not Sure 4. Disagree 5. Strongly disagree
5. The antibiotic is chosen according to the availability of the antibiotic more than the microbial cause of infection.	1. Strongly agree 2. Agree 3. Not Sure 4. Disagree 5. Strongly disagree
6. It is important to know the resistance rates in my workplace	1. Strongly agree 2. Agree 3. Not Sure 4. Disagree 5. Strongly disagree
7. International guidelines are more important than local policies in antibiotic prescription	1. Strongly agree 2. Agree 3. Not Sure 4. Disagree 5. Strongly disagree
8. Some antibiotics must be ordered only by a qualified senior physician	1. Strongly agree 2. Agree 3. Not Sure 4. Disagree 5. Strongly disagree
9. In all cases where antibiotics are dispensed, it is important that patients are advised about complying with the treatment.	1. Strongly agree 2. Agree 3. Not Sure 4. Disagree 5. Strongly disagree

10. The use of antibiotics in livestock animals is an important cause of the appearance of new resistance to pathogenic agents in humans	1. Strongly agree 2. Agree 3. Not Sure 4. Disagree 5. Strongly disagree

SECTION 4 .PRACTICES WHILE USING ANTIMICROBIALS

1. .How frequently do you prescribe antibiotics in your clinical practice?

★ once daily or more ★ once weekly

★ 1–2 times per week

★ 3–5 times per week

★ once monthly

2. .What percentage of your antibiotic prescriptions are for:

★ Respiratory Tract Infections (RTIs)

★ Urinary Tract Infections (UTIs)

★ Skin and Soft Tissue Infections (SSTIs)

- ★ ☐ Other (please specify)

3. .What diagnostic methods do you commonly use to determine whether antibiotics are necessary for a patient's condition? Select all that apply.

- ★ ☐ Clinical assessment
- ★ ☐ Laboratory tests (e.g., cultures, blood tests)
- ★ ☐ Imaging studies
- ★ ☐ Other (please specify)

4. To what extent does rapid diagnostic testing influence your decision to prescribe antibiotics?

- ★ ☐ Significantly
- ★ ☐ Moderately
- ★ ☐ Minimally
- ★ ☐ Not at all

5. What factors influence your decision to prescribe antibiotics? Select all that apply

- ★ ☐ Patient symptoms
- ★ ☐ Previous patient response to antibiotics
- ★ ☐ Patient preference
- ★ ☐ Local antibiotic resistance patterns

6. I consult with infectious diseases experts to prescribe of broad-spectrum antibiotics

- ☐ Never
- ☐ Sometime
- ☐ Often
- ☐ Always

7. I educate people on the use of antibiotics and resistance related issues whenever I can

- ☐ Never
- ☐ Sometime
- ☐ Often
- ☐ Always

8. I participate in antibiotic awareness campaigns to promote the optimal use of antibiotics

- ☐ Never
- ☐ Sometime
- ☐ Often
- ☐ Always

9. I have attended Continuing Professional Development (CPD) education on antibiotic use and resistance topics

★ Yes

★ No

10. I have taken courses to improve my knowledge of antimicrobial resistance and antibiotic use

★ Yes

★ No

Precision medicine moderator guide and focus group questions (Yeh et al., 2020)

1. What types of things have you heard about precision medicine?

a. Where have you heard these things?

b. From who?

According to the National Institutes of Health, precision medicine is an approach to disease treatment and prevention that seeks to maximize effectiveness of therapies by taking into account an individual's genes, environment, and lifestyle. 2. What do you think about precision medicine(PM)?

3. How do you think precision medicine will affect the health care you receive? How do you think PM will affect health care overall?
4. How do you think precision medicine will affect your daily life?
5. How accessible do you think precision medicine is now? How accessible will it be in the future?
6. Who do you think will benefit the most from precision medicine?
7. Do you have any concerns about precision medicine? What types of information would be helpful to address these concerns?
8. Do you think that using precision medicine more would require doing less of something else in medical care? If so, what would you be willing to give up in order to get more use of precision medicine?
9. Who do you think would be the best person or organization to provide information about precision medicine to you and your community? How would you like to learn about precision medicine?
