# Supplementary material for: Navigating Antimicrobial Resistance Insights: An In-Depth Analysis of Healthcare Providers’ Knowledge, Attitudes, and Practices, with an Emphasis on Precision Medicine in Pakistan
Source: Antibiotics (Basel). 2025 Dec 18;14(12):1281. doi: 10.3390/antibiotics14121281 (PMC12729868; doi:10.3390/antibiotics14121281)
Supplement: Supplementary file 1 [file antibiotics-14-01281-s001.zip › antibiotics-3883016-supplementary2.pdf]

Supplementary material

Regarding knowledge about antibiotics, the majority of participants correctly identified that antibiotics are only effective against bacterial infections (87.5%) and recognized that some bacteria may be resistant to certain antibiotics (68%). However, misconceptions persisted, with 13.8% incorrectly associating antibiotic resistance with the body's resistance to antimicrobials and 59.1% believing that newer or more expensive antibiotics are more effective. There was a notable gap in recognizing all factors contributing to antibiotic resistance, such as inadequate hand washing (12.3%) and lack of immunization campaigns (22.2%). Awareness of specific antibiotics and resistance spread was variable, with 71.7% knowing metronidazole targets anaerobes, but only 45.3% understanding how easily resistant bacteria spread.

Regarding attitudes towards antimicrobial use and resistance, majority of HCPs (60.1%) recognize antimicrobial resistance as a significant public health issue in Pakistan. Many believe that limiting antibiotic use could impair patient care (29.9%) and that unnecessary use of antibiotics can cause patient harm (66.2%). To address these concerns, 59.1% advocate for the establishment of courses on rational antibiotics use. Additionally, a considerable proportion of respondents (54.9%) prioritize antibiotic selection based on availability rather than the microbial cause of infection, and 43% stress the importance of knowing resistance rates in their workplace. Nearly half (45.4%) support involving senior health care professionals in antibiotic prescription and emphasize the need for patient compliance (48.2%) to combat AMR. Many also recognize the role of antibiotic use in livestock (34.35%), highlighting the need for broader antimicrobial stewardship including agriculture.

In terms of practices, most professionals prescribe antibiotics frequently, with 56.7% doing so daily or more often. Diagnostic methods primarily involve clinical assessment and laboratory tests (75.1% and 75.7%, respectively), and 46.6% report that rapid diagnostic testing significantly impacts their prescribing decisions. Additionally, while many consult with infectious disease experts and follow standard treatment guidelines, 47.9% acknowledge the influence of multinational companies on antibiotic prescribing. (For detailed responses refer to supplementary tables 6,7 and 8)

Table: Knowledge of healthcare professionals regarding antimicrobial use and resistance

**Knowledge Item (Correct Response)**	**N**	**%**
Antibiotics are only effective against bacterial infections (yes)	287	87.5
Antibiotics are selective and may not work against certain bacteria (yes)	221	68
Conditions that can be treated with antimicrobials		
UTIs,	311	95.7
skin or wound infections	291	89.5
gonorrhea	224	68.9
Antibiotic resistance occurs when your body becomes resistant to antimicrobials and they no longer work as well (No)	45	13.8
The efficacy of antibiotics is higher if it is newer or of higher price (No)	192	59.1
The most contributory towards antimicrobial resistance are		
Over usage of antimicrobials by prescriptions	255	75.8
Over usage of antimicrobials without prescriptions	277	85.2
Errors in medical prescriptions (dose, duration of use, and choice)	168	51.7
Non-compliance of patients with prescribed treatment	216	66.5
Patient pressure for antimicrobial prescriptions	159	48.9
Inadequate hand washing	40	12.3
Lack of immunization campaigns	72	22.2
Lack of new antimicrobial drugs	71	21.8

Use of antimicrobials as growth promoters in animals	48	14.8
Antibiotics with best activity against anaerobes (Metronidazole)	233	71.7
Antibiotic resistant bacteria spread easily from person to person (Yes)	143	45.3
Correct duration of prescribing antibiotics for an uncomplicated bacterial sinus infection	148	46.8

Table: Attitude of healthcare professionals towards antimicrobial use and resistance

**Statement**	**Strongly agree**		**Agree**		**Neutral**		**Disagree**		**Strongly disagree**	
	N	%	n	%	N	%	N	%	n	%
Antimicrobial resistance is a serious public health issue in Pakistan.	197	60.3	12 2	37. 5	5	1.5	2	0.6	1	0.3
By limiting the use of antibiotics, good patient care would be impaired.	51	15.7	98	29. 9	5 9	18	95	29	24	7.3
I believe that prescribing antimicrobials does not cause any damage when patients don't need them.	28	8.5	38	11. 6	4 4	13. 4	11 7	35.7	10 0	30. 5
We need to establish courses on rational use of antimicrobials	194	59.1	11 0	33. 5	1 9	5.8	4	1.2	0	**0**
The antibiotic is chosen according	72	22	10	32.	4	13.	76	23.2	27	8.2

to the availability of the antibiotic more than the microbial cause of infection.			8	9	4	4				
It is important to know the resistance rates in my workplace	141	43	157	47.9	25	7.6	3	0.9	1	0.3
International guidelines are more important than local policies in antibiotic prescription	108	32.9	99	30.2	57	17.4	53	16.2	10	3
Some antibiotics must be ordered only by a qualified senior physician	149	45.4	137	41.8	23	7	16	4.9	2	0.6
In all cases where antibiotics are dispensed, it is important that patients are advised about complying with the treatment.	158	48.2	137	41.8	18	5.5	15	4.6	0	0
The use of antibiotics in livestock animals is an important cause of the appearance of new resistance to pathogenic agents in humans	60	18.3	113	34.5	105	32	38	11.6	12	3.7

Table: Practices of healthcare professionals towards antimicrobial use and resistance

**Practice Item**	**N**	**%**
Frequency of antibiotic prescription		
• once daily or more	186	56.7
• once weekly	75	22.9
• 1–2 times per week	34	10.4
• 3–5 times per week	9	2.7

• once monthly	23	7.0
Diagnostic methods commonly used to determine antibiotic need for a patient's condition<br>• Clinical assessment<br>• Laboratory tests (e.g., cultures, blood tests)<br>• Imaging studies<br>• Other	 244<br>246<br>69<br>1	 75.1<br>75.7<br>21.2<br>0.3
Extent to which rapid diagnostic testing influence your decision to prescribe antibiotics<br>Significantly<br>Moderately<br>Minimally<br>Not at all	153<br>141<br>26<br>8	46.6<br>43.0<br>7.9<br>2.4
I consult with infectious diseases experts to prescribe of broad-spectrum antibiotics<br>Always<br>Often<br>Sometimes<br>Never	55<br>122<br>101<br>53	15.5<br>37.2<br>30.8<br>16.2
I educate people on the use of antibiotics and resistance related issues whenever possible<br>Always<br>Often<br>Sometimes<br>Never	123<br>133<br>66<br>5	37.5<br>40.5<br>20.1<br>1,5

Doctors get influenced by multinational companies to write antibiotics<br>Always<br>Often<br>Sometimes<br>Never	46<br>157<br>101<br>123	14<br>47.9<br>30.8<br>7
I follow the standard treatment guidelines when dealing with infectious diseases<br>Always<br>Often<br>Sometimes<br>Never	105<br>148<br>65<br>9	32<br>45.1<br>19.8<br>2.7
The frequency of reviewing the decision to prescribe antibiotics with a senior colleague?<br>Always<br>Often<br>Sometimes<br>Never	68<br>77<br>164<br>18	20.7<br>23.5<br>50<br>5.5
